# Clinical Utility of Beck Anxiety Inventory in Clinical and Nonclinical Korean Samples

**DOI:** 10.3389/fpsyt.2018.00666

**Published:** 2018-12-04

**Authors:** Hyeonju Oh, Kiho Park, Seowon Yoon, Yeseul Kim, Seung-Hwan Lee, Yoon Young Choi, Kee-Hong Choi

**Affiliations:** ^1^Department of Psychology, Clinical and Counseling Psychology, Korea University, Seoul, South Korea; ^2^Department of Adolescent Counseling, Hanyang Cyber University, Seoul, South Korea; ^3^Clinical Emotion and Cognition Research Laboratory, Inje University, Goyang, South Korea; ^4^Department of Psychiatry, Ilsan Paik Hospital, College of Medicine, Inje University, Goyang, South Korea

**Keywords:** beck anxiety inventory, diagnostic utility, psychometric property, anxiety, evidence-based assessment

## Abstract

Despite the prominent use of the Beck Anxiety Inventory (BAI) in primary healthcare systems, few studies have confirmed its diagnostic utility and psychometric properties in non-Western countries. This study aims to clarify the clinical utility of the BAI as a screening tool for anxiety disorders according to DSM-IV criteria, based on blind recruitment and diagnostic interviews of both clinical and non-clinical participants in the Korean population. A total of 1,157 participants were involved in the final psychometric analysis, which included correlational analysis with other anxiety and depression self-report measures and mean score comparison with the Beck Depression Inventory (BDI). ROC analysis and calculation of positive and negative predictive values were conducted to examine diagnostic utility. The BAI was found to have high correlations with depression-related self-report measures (0.747–0.796) and moderate to high correlations with anxiety-related self-report measures (0.518–0.776). The ROC analysis failed to provide cutoff scores with adequate sensitivity and specificity for identifying participants with anxiety disorders (85.0% sensitivity, 88.1% specificity, and 92.8% AUC). The comparison of BAI and BDI mean scores for different diagnostic groups revealed that BAI and BDI scores were higher in the depressive or anxiety disorders group than in the non-clinical group. However, BAI mean score was not higher for the anxiety-only group than the depression-only group. Our data supports the BAI reliability and validity as a tool to measure the severity of general anxiety in clinical and non-clinical populations; however, it fails to capture the unique characteristics of anxiety disorders that distinguish them from depressive disorders. Further clinical implications of the BAI based on these results and some limitations of the study are discussed.

## Introduction

The Beck Anxiety Inventory (BAI) is a prominent screening and outcome research tool for measuring the anxiety (1), and is validated in a number of languages, including German, French, Chinese, Spanish, Persian, Nepal, Icelandic, and others (2–7). Strong psychometric evidences have been established in diverse samples, including diverse clinical samples mixed psychiatric patients (8–14), panic disorder with and without agoraphobia (15), adolescent psychiatric patients (13, 16, 17), and older adult psychiatric patients (18) to non-clinical samples (19–21). The BAI requires about 5–10 min to administer (about 10 min for oral administration), and <5 min to interpret the scores. Among counselors in primary health care settings, BAI is reported to be the ninth most commonly used tools in the United States (22), owing to the advantages in cost-effectiveness and brevity in the application procedure. According to the meta-analysis of BAI (*k* = 117), BAI was reported to manifest an excellent internal consistency in clinical (0.91) and non-clinical sample (0.91) and a good test-retest reliability in clinical (0.66) and non-clinical (0.65) (1).

This 21-item self-report questionnaire was originally developed to assess clinical anxiety (i.e., an excess of normal anxiety resulting in significant distress and impairment of functioning (23, 24), differentiated from normal anxiety (i.e., an adaptive emotional responses to danger or threat, (25, 26), as well as the unique aspects of anxiety disorders that are assumed to differ from those of depressive disorders (27). While depression is defined as the experience of being sad, gloomy, and empty which is typically associated with events experienced in the past and decreased autonomic activity, anxiety is defined as feelings of fear and tension, and apprehension which is usually related to anticipation of future events and activation of autonomic nervous system (28).

However, some issues remain regarding the BAI's discriminant validity against depressive disorders. Distinguishing anxiety and depressive disorders through self-report measures has been the subject of debate due to a high rate of comorbidity or the possibility of a single, shared underlying mechanism, such as negative affect (28). Although Beck's original studies report significantly higher BAI mean scores for patients with anxiety disorders compared to those with depressive disorders (9, 27), other studies fail to replicate the results (29). In one study conducted in Korean sample, correlation coefficients of BAI with other depression assessments such as BDI and PHQ-9 are found to be even higher than other anxiety assessment tools (30). The ambiguity found in the mean scores difference of BAI and correlation sizes with other anxiety and depression measures questions the utility of BAI as measuring the general anxiety that are distinguishable from depression as Beck et al. (27). Because various anxiety and depression tools aim to assess the same construct with heterogeneous factors, more diverse assessments must be incorporated into an analysis to provide a comprehensive outlook on the divergent and convergent validity of the BAI.

Another issue concerns the BAI's clinical utility as an anxiety screening tool and a measurement of severity in primary care settings. Although the BAI was not originally developed as a diagnostic tool, it is essential to examine the degree of its diagnostic reliability and its score distribution in a sample before it can be utilized as a tool in anxiety screening, to track symptom changes, or as an outcome measure based on severity measurements. To date, 11 studies explored the diagnostic validity of BAI, optimal cutoff scores ranging from 7 to 26 depending on the diversity of studied samples (1). As the diagnostic cutoff score can be varying across the ethnicity and cultural background behind the research setting, undertaking the diagnostic validity study in a new ethnic sample can benefit the existing literature. Negative and positive predictive powers are critical sources for determining the clinical utility of a screening tool, especially for disorders in low prevalence rate (12). Providing such information would thus help researchers to decide optimal BAI cutoff scores for their individual purposes.

The BAI is a prominent anxiety assessment tool in Korea, with uses ranging from intervention outcome measures to mechanism studies. According to the epidemiological survey of mental disorders in Korea conducted by ministry of health and welfare, the estimated lifetime prevalence rate of anxiety disorders for Korean adults was 9.3% (male 6.7%, female 11.7%) and 1-year prevalence rate of anxiety disorders for Korean adults was 5.7% (male 3.8%, female 7.5%) (31). It was suggested that a social stigma against mental illness and inability to recognize the need of treatment may contribute to lower prevalence rates in Korea than that of Western countries, and hence the need to develop screening tools to identify the targets who need treatment was emphasized. Thus, far, the factor structure of BAI has been heterogeneously reported across studies from 2 factors to 4 factors (32, 33), which are comparable to the BAI literature conducted in other languages (1). Internal consistency and test-retest reliability of the Korean BAI have been reported as 0.91–0.93 and *r* = 0.84, respectively (30, 32, 34). Before publication of the formal translated version of the BAI by Korea Psychology Co., Ltd, which underwent several back-translation processes, all previous studies except but the most recent study (30) were based on a version of the BAI independently translated by Kwon (34). The study with newly translated version reported moderate correlation of the BAI with STAI-S, STAI-T, and PHQ-9, with excellent reliability (30). However, this was based on a stratified sample of community-dwelling people; no literature is yet available to determine the clinical utility of the BAI in Korean psychiatric populations.

Using unbiased clinical and non-clinical samples, this study aims to (1) determine the divergent and convergent validity of the BAI compared to an extended set of proxy self-report questionnaires designed to measure depression and anxiety symptoms in Korean clinical and non-clinical samples; (2) provide a comparison of BAI severity scores in anxiety disorder only, depressive disorder only, and comorbid anxiety and depression populations; and (3) reconsider the clinical utility of the BAI by determining cutoff scores and calculating specificity and sensitivity as well as positive and negative predictive power using Korean anxiety disorder base rates.

## Materials and Methods

### Subjects

The current study was conducted using data from the Development and Validation of the Korean Depression and Anxiety Scales Project, collected from September 2016 to July 2018. The ethical approval was made by Korea University Institutional Review Board [1040548-KU-IRB-15-92-A-1(R-A-1)(R-A-2)(R-A-2)] and Inje University Medical Institutional Review Board (ISPAIK 2015-05-221-009). Participant sampling for the current study was undertaken at three different sites, a university-affiliated mental health institute and two general hospitals. An aggregate effect-size score for BDI-II and BAI in 109 studies published since 1993 is *r* = 0.59 [95% CI [0.58, 0.60], *n* = 28,533] (1). In order to get 0.95 power for this effect-size, 76 participants were required in each group for the analysis (G^*^Power 3.1) (35, 36). All participants were voluntarily enrolled in the study and consecutively sampled until the sample size of participants diagnosed with anxiety and depressive disorders exceed the required number. The risk of bias in sampling and assessment procedures was evaluated using the QUADAS-2 tool (Table 1). The one inclusion criterion was (1) age over 18 years. Exclusion criteria included individuals with (1) inappropriate responses, (2) history of surgery, and (3) presence of other major disorders. Participants were provided with a detailed account of the current study, signed an informed consent form, and then proceeded with the self-report questionnaires and diagnostic interview. Supervision of the self-report questionnaires and interviews were conducted independently by different investigators to ensure blind sampling; researchers who conducted the structured psychiatric interviews were blinded to the participants' scores on depression- or anxiety-related self-questionnaires. Psychiatric diagnosis was confirmed by the Mini-International Neuropsychiatric Interview (MINI), plus version (37, 38). A total of 1,196 participants were initially recruited. Thirty-nine participants from one of the general hospitals were excluded from the final analysis based on unfinished self-report questionnaires (26 participants), refusal to participate in the interview (11 participants), age under 18 years (1 participant), and Japanese nationality (1 participant), resulting in 1,157 participants for statistical analysis. Participant demographics are shown in Table 2.

**Table 1:** Evaluation of the Current Study in QUADAS-2 Domains and Signaling Questions.

**Domains of Risk of Bias**	**Signaling Questions**	**Current study performance**
Patient selection Could the selection of patients have introduced bias?	Was a consecutive or random sample of patients enrolled? Was a case-control design avoided? Did the study avoid inappropriate exclusions?	Low Yes –consecutive Yes Yes
Index test Could the conduct or interpretation of the index test have introduced bias?	Were the index test results interpreted without knowledge of the reference standard? If a threshold was used, was it pre-specified?	Low Yes Not used
Reference standard Could the reference standard, its conduct, or its interpretation have introduced bias?	Is the reference standard likely to correctly classify the target condition? Were the reference standard results interpreted without knowledge of the results of the index test?	Low Yes (MINI Plus 5.0.0) Yes
Flow and timing Could the patient flow have introduced bias?	Was there an appropriate interval between the index test and the reference standard? Did all patients receive the same reference standard? Were all patients included in the analyses?	Yes (conducted on the same day) Yes No (reasons for exclusion reported)

**Table 2:** Participant Characteristics.

	***M***	***SD***
Age	37.38	14.64
Education (years)	14.63	2.98
	**N**^a^	**%**
**GENDER**		
Male	381	33
Female	772	67
**MARITAL STATUS**		
Never married	652	57.1
Married	444	38.9
Divorced	21	1.8
Widowed	24	2.1
**DIAGNOSIS**		
Major depressive disorder	105	9.1
Depressive disorder	191	16.5
Generalized anxiety disorder	97	8.4
Anxiety disorder	223	19.3
Bipolar disorders I & II	63	5.4
Other psychiatric disorders	193	16.7
No psychiatric disorder	712	61.5

a *Total may be < 1,157 due to missing data*.

### Instruments

#### Mini-International Neuropsychiatric Interview Plus Version 5.0.0 (MINI)

The MINI, which was utilized as a reference standard in the present study, is a structured interview tool developed for diagnosis of major Axis I mental disorders from the tenth revision of the International Classification of Diseases (ICD-10) and the fourth edition of the *Diagnostic and Statistical Manual of Mental Disorders* (37). The present study adopted the Korean version of the MINI, which has a good level of diagnostic accuracy (38). Clinical psychology graduate students and psychiatry fellows conducted the interviews, and their diagnostic decisions were supervised by licensed psychologists and psychiatrists. An excellent inter-rater reliability on the MINI diagnoses was found (ICC = 0.92).

#### Penn State Worry Questionnaire (PSWQ)

The PSWQ is a self-report questionnaire developed to identify excessive and uncontrollable pathological worries, consisting of 16 items rated on a 5-point Likert scale from 1 (*not at all typical of me*) to 5 (*very typical of me*) (39). Multiple studies in both clinical and non-clinical samples have manifested high internal consistency and convergent and criterion-related validity. Although the PSWQ is not a diagnostic scale, it is highly sensitive to concerns related to generalized anxiety disorder (GAD), and the cutoff score of 65 is reported to have optimal sensitivity and specificity to distinguish patients with GAD from patients with other anxiety disorders, such as social anxiety disorder (40). The Korean version of the PSWQ demonstrates an internal consistency coefficient of 0.93 and test-retest reliability of *r* = 0.90 for a 4-week period (41).

#### Generalized Anxiety Disorder 7-Item Scale (GAD-7)

The GAD-7 was developed to screen and assess the severity of GAD symptoms. The tool includes 7 items rated on a 4-point Likert scale from 0 (*not at all*) to 3 (*nearly every day*) (42), and is widely used in primary healthcare settings due to its high efficiency. A Korean version of the GAD-7 was validated for patients with migraines (43) and epilepsy (44), demonstrating high internal reliability and respective cutoff scores of 5 and 6, with adequate sensitivity and specificity.

#### Beck Anxiety Inventory (BAI)

The BAI is a self-report assessment of anxiety symptoms, initially developed to differentiate between anxiety and depression. The BAI consists of 21 items rated on a 4-point Likert scale from 0 (*not at all*) to 3 (*severely*). The first validation study of BAI reports 0.93 for internal consistency and 0.84 for test-retest reliability (34). There is no study available reporting a cutoff score of BAI in any sample in Korea. A copyrighted version distributed by Pearson Assessments was newly translated (30) and adopted in our study.

#### Beck Depression Inventory-II (BDI-II)

The BDI is a self-report depression scale developed by Beck et al. to measure affective, cognitive, motivative, and physiological aspects of depression, and is widely used in both research and clinical settings (27). The BDI-II, published subsequently, introduced changes in domain and duration cues for measurement (45). The BDI-II consists of 21 items rated on a 4-point Likert scale from 0 to 3. In Korea, several independent groups undertook validation of the BDI-II (46–48). Lim et al. (47) report a cutoff score of 18 with 85.0% sensitivity, 88.1% specificity, and 92.8% AUC, which demonstrates high diagnostic utility (47). The BDI-II was used as a crucial reference in the present study to determine the diagnostic and clinical utility of the BAI.

#### Center for Epidemiologic Studies Depression Scale (CES-D)

The CES-D is a self-report assessment tool to accessibly measure depression in the general population using selected items from several validated depression scales such as the BDI, the Zung Self-Rating Depression Scale, and the Minnesota Multiphasic Personality Inventory Depression Scale (49). The tool consists of 20 items that measure affective, physical, and interpersonal symptoms on 4-point Likert scale from 0 (*rarely or none of the time*) to 3 (*most or all of the time*). Korean versions of the CES-D were independently adapted and validated by multiple groups, followed by the development of an integrated version, the K-CES-D (50). Cho and Kim (51) report a cutoff score of 25 points for major depressive disorder patients in Korea, 91.3% sensitivity, 78.8% specificity, and 91.4% AUC (51).

#### Patient Health Questionnaire 9-Item Depression Module (PHQ-9)

The PHQ-9 is a self-report questionnaire developed to screen for and measure the severity of depression in primary healthcare settings (52). It consists of 9 items required for diagnosis of major depressive disorder on a 4-point Likert scale from 0 (*not at all*) to 3 (*nearly every day*) and 1 item that measures the severity of daily life difficulty. The Korean version of the PHQ-9 was validated by various groups (53–55). The most recent study indicates a cutoff score of 9 points, 88.5% sensitivity, 94.7% specificity, and 97.6% AUC (55).

#### Anxiety Sensitivity Index-3 (ASI-3)

The ASI-3 is a revised version of the ASI (56), a self-report questionnaire to measure anxiety sensitivity, the degree to which physiological arousal is interpreted as a threat (57). The ASI-3 consists of 18 items rated on a 5-point Likert scale from 0 (*very little*) to 4 (*very much*). In Korea, Lim and Kim (58) have validated the tool, reporting an internal consistency of 0.87, and identified three low-order domains using factor analysis (physical, social, and cognitive concerns) (58).

#### Albany Panic and Phobia Questionnaire (APPQ)

The APPQ is a self-report questionnaire developed to assess and evaluate activities that elicit interoceptive fear, agoraphobia, and social phobia (59). The scale consists of 27 items rated on a 9-point Likert scale from 0 (*no fear*) to 8 (*extreme fear*). A Korean validation version of the APPQ reports an internal consistency of 0.95 and a test-retest reliability of 0.77 (60).

### Procedure

This study utilized the MINI Plus version 5.0.0 as a diagnostic reference regarding participants' psychological disorders. Interviewers arrive at diagnostic conclusions by following the tool's instructions, and additional inquiries are made to clarify individuals' clinical features. In this study, the interviewers who conducted the interviews were blinded to the results of the self-report questionnaires to ensure that the diagnostic process was not influenced by the self-report results. The diagnostic interviews were conducted by trained graduate students and one certified clinical psychologist, and final diagnoses were confirmed through case conferences.

The IBM Statistics 23 software package was used for calculating Pearson's *r* effect-size estimates of proxy scales as well as ROC curve analysis. Positive predictive value (PPV) and negative predictive value (NPV) were calculated using the lifetime prevalence (9.3%) of anxiety disorder as assessed by the Ministry of Health and Welfare in Korea (31). BAI mean scores for depressive disorder only group, anxiety disorder only group, and comorbid group were also calculated. For the BAI and BDI mean score comparison, the sample was divided into five groups: anxiety disorder only group (i.e., disorders under anxiety disorder category under DSM-IV, with no other comorbid disorder category), depressive disorder only group (i.e., disorders under depressive disorder category under DSM-IV, with no other comorbid disorder category), comorbid group (i.e., having both categories of anxiety and depressive disorder category under DSM-IV), other psychiatric patients group (i.e., obsessive compulsive disorder, eating disorder, and other psychiatric patients without anxiety or depressive disorders) and healthy control group with no psychiatric diagnosis.

## Results

### Participant Demographics and Diagnoses

A total of 1,157 individuals participated in this study. As confirmed by the MINI, 9.1% of participants were diagnosed with major depressive disorder, 16.5% with any type of depressive disorder, 8.4% with GAD, 19.3% with any type of anxiety disorder, and 5.4% with bipolar disorders. The number of female participants 772 (66.7%) was almost twice that of male participants. Participants ranged in age from 19 to 84.

### Correlation Analysis

Our data analysis manifested a Cronbach's alpha of 0.956, which was higher than any other studies reporting internal consistency. The item-total correlations ranged from 0.358 to 0.744. Correlation analysis with proxy measures of depression and anxiety symptoms yielded heterogeneous results. The BAI showed moderate to high correlation with anxiety-related scales, and high correlation with depression-related scales (Table 3).

**Table 3:** Correlation with Proxy Scales.

	**BAI**
ASI^a^	0.724^**^
APPQ^b^	0.640^**^
GAD-7^c^	0.776^**^
PSWQ^d^	0.518^**^
BDI-II^e^	0.796^**^
CES-D	0.747^**^
PHQ-9	0.769^**^

a *238 participants*.

b *239 participants*.

c *1147 participants*.

d *1091 participants*.

e *1147 participants*.

** *p < 0.01*.

### Comparison of BAI and BDI Mean Scores Across Diagnoses

As expected, significant mean score differences were found between the anxiety disorder only group (*M* = 11.65, *SD* = 11.24) and the control group without any psychiatric diagnosis (*M* = 4.26, *SD* = 5.18, *p* < 0.0001). For both the anxiety disorder only and depressive disorder only group, BAI and BDI scores were higher than the non-clinical group. Interestingly, BAI scores were lower than BDI scores in the anxiety disorder only group. The comorbid group showed the highest mean scores for both BAI and BDI (Table 4).

**Table 4:** BAI and BDI Mean Scores for Depression and Anxiety Disorders.

	**BAI**			**BDI**		
	***N***	***M***	***SD***	***N***	***M***	***SD***
Depressive disorders only	81	15.12	10.878	81	22.75	11.565
Anxiety disorders only	93	11.90	10.676	93	18.53	11.061
Comorbid depression/anxiety	102	25.64	15.761	101	31.19	13.907
Other psychiatric disorders	166	9.69	9.988	165	16.39	10.854
Nonclinical	708	4.28	5.049	707	8.82	6.953
Total	1150	8.34	10.511	1147	13.65	11.534

### Diagnostic Utility of the BAI

ROC curve analysis for the anxiety disorders only group revealed an optimal cutoff score of 8, sensitivity of 0.75, and specificity of 0.745. This means that a score of 8 or higher will identify 75% of those with an anxiety disorder and exclude 74% of those without. We additionally calculated PPV (30.8%) and NPV (69.2%) for the scale using the lifetime prevalence of 9.3% (31). This indicates that only three out of every 10 people identified by the BAI as having an anxiety disorder actually do have the disorder. The sensitivity and specificity rates for each cutoff score are presented in Table 5.

**Table 5:** Specificity and Sensitivity for Anxiety Disorders at Selected Cutoff Scores.

**BAI cutoff score**	**Sensitivity**	**Specificity**
1	0.977	0.198
2	0.945	0.305
3	0.905	0.425
4	0.873	0.506
5	0.845	0.575
6	0.809	0.633
7	0.777	0.688
8	0.750	0.740
9	0.714	0.776
10	0.668	0.798
11	0.636	0.828
12	0.591	0.848
13	0.568	0.868
14	0.532	0.881
15	0.491	0.895
16	0.473	0.906
17	0.450	0.916
18	0.432	0.925
19	0.405	0.931
20	0.391	0.943
21	0.386	0.953
22	0.368	0.959
23	0.341	0.962
24	0.314	0.968
25	0.305	0.971
26	0.286	0.974
27	0.277	0.978
28	0.268	0.982
29	0.264	0.985
30	0.255	0.987
31	0.245	0.989
32	0.232	0.991
33	0.209	0.991
34	0.200	0.992
36	0.195	0.992
37	0.164	0.994
39	0.159	0.994
41	0.150	0.994
42	0.136	0.995
44	0.132	0.996
45	0.114	0.997
47	0.100	0.997
48	0.082	0.997
49	0.073	0.997
50	0.068	0.997
55	0.064	0.998
59	0.059	0.999
62	0.050	1.000
64	0.045	1.000

## Discussion

The present study illustrates the psychometric properties and diagnostic utility of the BAI in blind samples of clinical psychiatric and non-clinical Korean adult populations. The BAI demonstrated excellent internal reliability and significant difference in mean score between the anxiety disorder and non-clinical groups. However, the scale did not demonstrate adequate discriminant validity between anxiety and depressive disorders, nor diagnostic utility for anxiety disorders under DSM-IV.

Our correlational analyses and mean score comparisons showed that the BAI appears to possess good convergent validity with various anxiety measures such as the ASI-3, APPQ, and GAD-7, and discriminant validity for those with and without an anxiety disorder diagnosis. However, our data did not support divergent validity within samples for depressive disorders; the correlation of the BAI with depression-related scales were mostly found to be higher than with other anxiety-related scales in the Korean sample. Convergent validity with other anxiety scales was moderate to high, which is suitable considering that the various anxiety scales included in this study measure heterogeneous concepts of anxiety, such as worry, sensitivity to physiological anxiety, and fear responses to certain objects. ROC curve analysis revealed that none of the cutoff scores demonstrated adequate diagnostic sensitivity and specificity for screening participants with anxiety disorders from those without anxiety disorders, including both clinical and non-clinical participants.

The correlation of the BAI with depression scales was found to be slightly higher in our study than in previous literature (15, 61, 62). Whereas, the diagnostic criteria for depressive disorders are based on more homogenous and robust depressive symptomatology that span various depressive disorders differing in the duration, repetition, and severity of depressive episodes, anxiety disorders manifest considerable heterogeneity under DSM-5 categorization (29). For instance, severity and frequency in social anxiety disorder may be determined by the frequency of encountering threatening social contexts. Scales such as the ASI-3 isolate the social aspect of anxiety as a factor that may be more sensitive to social anxiety disorder than other disorders in the anxiety category in DSM-5. In contrast, a GAD patient with predominant and excessive worry may score high on worry-focused scales such as the PSWQ. The items in the BAI do not encapsulate these specificities, although it may successfully capture somatic or panic-related symptoms (12, 63).

Interestingly, in our Korean sample data, the depressive disorders only group showed higher BAI mean scores than the anxiety disorders only group. Previous studies have reported higher BAI mean scores for the anxiety disorders only group (27), or at least showed no significant difference between the two (29). We speculate that cross-cultural factors could have contributed to the high BAI mean score in the depressive disorder only group in our study. That is, because a majority of BAI items concern somatization (14 out of 21 items), it is possible that the Korean sample expressed more severe somatization symptoms. In fact, somaticizing depression is not a new concept in cross-cultural psychology (64). A number of Chinese studies indicate a tendency to deny depression and express it somatically, along with cultural terms such as neurasthenia and *shenjing shuairuo* (64, 65). A recent study in Korea that explores the factor structure of collapsed items of the BAI and BDI-II shows somatic anxiety and somatic depression as major factors of the two collapsed mood disorder assessments, and notes cognitive renderings of anxiety and depression as a main discriminant factor (66). Thus, the cultural prevalence of somaticized psychiatric symptoms may have obscured the discriminant validity of the BAI against the depressive disorder group.

None of the cutoff scores in our studies revealed acceptable diagnostic sensitivity and specificity. Whereas, the cutoff score of about 15 to 16 is suggested as an optimal score by the original study (27) as well as the meta-analysis of studies reporting cutoff scores (1), a cutoff score of 8 was found to be the most acceptable combination of sensitivity and specificity in our study. Considering that the maximum total BAI score is 63, this score may be too low to adequately distinguish between those with and without anxiety disorders. Positive Predictive Value (PPV) and Negative Predictive Value (NPV) illustrate the diagnostic utility of the BAI in a real setting: only two out of every 10 people identified with an anxiety disorder based on a cutoff score of 8 will actually have the disorder. However, such a low threshold may still have clinical utility in settings where detection of anxiety is of higher importance than actually having the disorder, such as in primary healthcare. Clinicians and researchers utilizing Korean samples may benefit from the cutoff score table, PPV, and NPV as references, selecting one according to their individualized settings and purpose for utilizing the tool.

An interesting speculation found in our study is that the somatization measured in BAI may contribute to the high BAI mean scores in Korean depression disorder patients. Since BAI is a criterion-based measure rather than a diagnosis-based to anxiety disorders, its utility could be highlighted in domain-based descriptions of symptomatology rather than in categorical diagnosis. We failed to find a cutoff score to distinguish anxiety disorder patients from healthy population with adequate sensitivity and specificity values. In addition, BAI is not an effective tool to discriminate patients with anxiety disorders and depressive disorders. It is therefore suggested to use a more sensitive tool in primary health care settings, due to the high possibility to incorrectly diagnose anxiety disorders.

Some limitations of the study should be noted. We aggregated the anxiety disorder group as a whole, despite the diagnostic variation across sub-categorical anxiety disorders. Future studies with larger sample sizes will allow for comparison of BAI mean scores among different types of anxiety disorders. Furthermore, considering that depression and anxiety disorders are highly comorbid, separating anxiety disorder only and depressive disorder only groups may lead to laboratory-bound results. This may have contributed to the lower BAI mean score in the anxiety disorder only group than was found in previous literature, which mainly studied anxiety disorders without separating other comorbidities. However, dividing patients into anxiety disorder only, depressive disorder only, comorbid, and general psychiatric groups may provide incremental validity by enhancing the accurate portrayal of mean score among pure anxiety and depression groups, as well as allowing important comparisons between diagnostic-based patient differences.

The main limitations of self-reported questionnaires include the risks of self-recalled bias and its inability to measure objective biological parameters. The utilization of neuropsychological tools such as the functional near-infrared spectroscopy (fNIRS), electroencephalography (EEG) or event-related potential (ERP) measures have arisen as new potentials in academic and clinical psychiatry (67–69). For instance, EEG studies focus on asymmetric hemispheric activity in depression and anxiety (68, 70), and frontal lobe abnormalities, particularly the decrease in bilateral frontotemporal oxygenation, have been found in unipolar depression using the verbal fluency test using fNIRS (71). The implementation of neurophysiological and neuropsychological instruments may shed a light on finding anxiety- or depression-specific biomarkers.

Another means to overcome the vulnerability to distortion or biases of retrospective retrievals of episodic memory is applying current mobile technology. It is possible to measure anxiety symptoms in a real-time using ecological momentary assessment (EMA) method, applying the measure several times a day. Moreover, to address the burden of answering 21 questions of BAI at each time point of application, computerized adaptive testing (CAT) technique could be one solution. For instance, Gibbons, Weiss (72) developed an anxiety tool “CAT-ANX” using this technique, which automatically selects and presents the most adequate questions from the item bank with 431 items. An average of 12 questions could measure the anxiety symptom that is highly correlated with the result with the entire 431 questions (*r* = 0.94). Coupled with a mobile device, CAT technique could serve as a useful means to realize real-time, longitudinal, easily distributable assessments of anxiety symptoms with relatively fewer demands on service receivers.

Since anxiety and depressive disorders are highly comorbid (73, 74), psychological or biological treatments share common targets and mechanisms such as high emotional avoidance, rumination (75), and suppression of negative emotions (76). Despite the common features, specific treatment recommendations have been made: treatments for depressive disorders include diverse psychotherapy [e.g., interpersonal psychotherapy (IPT), cognitive-behavioral therapy (CBT)], psychopharmacological treatment (e.g., SSRI, MAOIs), transcranial magnetic stimulation (77), electroconvulsive therapy (78) For treatments of anxiety disorders, exposure-based psychotherapies (79), benzodiazepine, SSRIs, and serotonin-norepinephrine reuptake inhibitors (SNRIs) are commonly used (80). In addition, in a future study, it should be investigated whether distinctive treatment recommendations after diagnosing anxiety or depressive disorders may produce differential outcomes.

Despite these limitations, the strength of this study is in its unbiased sample, which strictly followed a robust quality assessment method using the QUADAS-2 tool (81). Our study satisfied all four domains in the risk of bias criterion. In addition, this study is the first to examine the clinical utility and psychometric properties of anxiety disorders involving both clinical and non-clinical samples in Korea. The data sample we used is assumed to reflect a natural primary healthcare setting, which presents a mixture of clinical, sub-clinical and non-clinical individuals. Additionally, the results from ROC curve analysis are expected to serve as a valuable reference in selecting cutoff scores for both clinicians and researchers according to their particular research design or clinical needs. However, attentive consideration must be made before utilizing the BAI in decision-making or outcome measures, in light of its equivocal convergent and discriminant validity.

## Author Contributions

HO, YYC, S-HL, and K-HC contributed to the conception and design of the study. K-HC supervised the overall study process. KP and HO performed data analysis. HO wrote the first draft of the manuscript. KP, SY, YK, and HO contributed to acquisition of data. All authors contributed to manuscript revision, and have read and approved the submitted version.

### Conflict of Interest Statement

The authors declare that the research was conducted in the absence of any commercial or financial relationships that could be construed as a potential conflict of interest.

## References

[1] Bardhoshi G Duncan K Erford BT Psychometric meta-analysis of the english version of the beck anxiety inventory. J. Couns. Dev. (2016) 94:356–73. 10.1002/jcad.12090

[2] Kohrt BA Kunz R Koirala N Validation of the Nepali version of beck anxiety inventory. J Inst Med. (2007) 25:1–4.

[3] Kaviani H Mousavi A Psychometric properties of the persian version of beck anxiety inventory (BAI). Tehran Univ Med JTUMS Pub. (2008) 66:136–40.

[4] Magán I Sanz J García-Vera MP. Psychometric properties of a spanish version of the beck anxiety inventory (BAI) in general population. Spanish J Psychol. (2008) 11:626–40. 10.1017/S1138741600004637 18988448

[5] Freeston M Ladouceur R Thibodeau N Gagnon F Rheaume J. The beck anxiety inventory. Psychometric properties of a French translation. L'Encephale (1994) 20:47–55. 8174510

[6] Sæmundsson BR Þ*órsdóttir F Kristjánsdóttir H Ólason DÞ* Smári J Sigurð*sson JF Psychometric properties of the icelandic version of the beck anxiety inventory in a clinical and a student population. Eur J Psychol Assess. (2011) 27:133–41. 10.1027/1015-5759/a000059

[7] Cheng SKW Wong CW Wong KC Chong GSC Wong MTP Chang SSY A study of psychometric properties, normative scores, and factor structure of the beck anxiety inventory–the Chinese version. Chin J Clin Psychol. (2002) 10:4–6.

[8] Beck AT Steer RA Carbin MG Psychometric properties of the beck depression inventory: twenty-five years of evaluation. Clin. Psychol. Rev. (1988) 8:77–100. 10.1016/0272-7358(88)90050-5

[9] Steer RA Ranieri WF Beck AT Clark DA Further evidence for the validity of the beck anxiety inventory with psychiatric outpatients. J Anxiety Disord. (1993) 7:195–205. 10.1016/0887-6185(93)90002-3

[10] Hewitt PL Norton GR The beck anxiety inventory: a psychometric analysis. Psychol Assess. (1993) 5:408 10.1037/1040-3590.5.4.408

[11] Steer RA Clark DA Beck AT Ranieri WF. Common and specific dimensions of self-reported anxiety and depression: a replication. J Abnorm Psychol. (1995) 104:542. 10.1037/0021-843X.104.3.542 7673579

[12] Leyfer OT Ruberg JL Woodruff-Borden J. Examination of the utility of the beck anxiety inventory and its factors as a screener for anxiety disorders. J Anxiety Disord. (2006) 20:444–58. 10.1016/j.janxdis.2005.05.004 16005177

[13] Steer RA Kumar G Ranieri WF Beck AT. Use of the beck anxiety inventory with adolescent psychiatric outpatients. Psychol Rep. (1995) 76:459–65. 10.2466/pr0.1995.76.2.459 7667457

[14] Fydrich T Dowdall D Chambless DL Reliability and validity of the beck anxiety inventory. J Anxiety Disord. (1992) 6:55–61. 10.1016/0887-6185(92)90026-4

[15] de Beurs E Wilson KA Chambless DL Goldstein AJ Feske U. Convergent and divergent validity of the beck anxiety inventory for patients with panic disorder and agoraphobia. Depress Anxiety (1997) 6:140–6. 10.1002/(SICI)1520-6394(1997)6:4<140::AID-DA2>3.0.CO;2-G 9559283

[16] Osman A Hoffman J Barrios FX Kopper BA Breitenstein JL Hahn SK. Factor structure, reliability, and validity of the beck anxiety inventory in adolescent psychiatric inpatients. J Clin Psychol. (2002) 58:443–56. 10.1002/jclp.1154 11920696

[17] Jolly JB Aruffo JF Wherry JN Livingston R The utility of the beck anxiety inventory with inpatient adolescents. J Anxiety Disord. (1993) 7:95–106. 10.1016/0887-6185(93)90008-9

[18] Kabacoff RI Segal DL Hersen M Van Hasselt VB. Psychometric properties and diagnostic utility of the beck anxiety inventory and the state-trait anxiety inventory with older adult psychiatric outpatients. J Anxiety Disord. (1997) 11:33–47. 10.1016/S0887-6185(96)00033-3 9131880

[19] Borden JW Peterson DR Jackson EA The beck anxiety inventory in nonclinical samples: initial psychometric properties. J Psychopathol Behav Assess. (1991) 13:345–56. 10.1007/BF00960446

[20] Osman A Barrios FX Aukes D Osman JR Markway K The beck anxiety inventory: psychometric properties in a community population. J Psychopathol Behav Assess. (1993) 15:287–97. 10.1007/BF00965034

[21] Creamer M Foran J Bell R. The beck anxiety inventory in a non-clinical sample. Behav Res Ther. (1995) 33:477–85. 10.1016/0005-7967(94)00082-U 7755538

[22] Peterson CH Lomas GI Neukrug ES Bonner MW Assessment use by counselors in the United States: implications for policy and practice. J Couns Dev. (2014) 92:90–8. 10.1002/j.1556-6676.2014.00134.x

[23] Emilien G Dinan T Lepola UM Durlach C Normal and pathological anxiety. In: Anxiety Disorder. Basel: Birkhäuser (2002). p. 1–30. 10.1007/978-3-0348-8157-9_1

[24] American Psychiatric Association Diagnostic and Statistical Manual of Mental Disorders (DSM-5®). Washington, DC: American Psychiatric Pub (2013).

[25] Epstein S The nature of anxiety with emphasis upon its relationship to expectancy. In: Anxiety; Current Trends in Theory and Research, New York, NY: Academic Press (1972). p. 291–337. 10.1016/B978-0-12-657402-9.50007-7

[26] Öhman A Fear and Anxiety as Emotional Phenomena: Clinical Phenomenology, Evolutionary Perspectives, and Information-Processing Mechanisms, New York, NY: Guilford Press (1993).

[27] Beck AT Epstein N Brown G Steer RA. An inventory for measuring clinical anxiety: psychometric properties. J Consul Clin Psychol. (1988) 56:893–7. 10.1037/0022-006X.56.6.893 3204199

[28] Feldman LA. Distinguishing depression and anxiety in self-report: evidence from confirmatory factor analysis on nonclinical and clinical samples. J Consult Clin Psychol. (1993) 61:631–8. 10.1037/0022-006X.61.4.631 8370858

[29] Muntingh ADT van der Feltz-Cornelis CM van Marwijk HWJ Spinhoven P Penninx BWJH van Balkom AJLM. Is the beck anxiety inventory a good tool to assess the severity of anxiety? A primary care study in The Netherlands study of depression and anxiety (NESDA). BMC Fam Pract. (2011) 12:66. 10.1186/1471-2296-12-66 21726443 PMC3224107

[30] Lee HK Lee EH Hwang ST Hong SH Kim JH Psychometric properties of the beck anxiety inventory in the community-dwelling sample of Korean adults. Korean J Clin Psychol. (2016) 35:822–30. 10.15842/kjcp.2016.35.4.010

[31] Ministry of Health and Welfare Seoul: Statistics Korea (KR). Korean. Available online at: https://meta.narastat.kr/metasvc/index.do?confmNo=117050 (cited November 27, 2018).

[32] Yook S Kim Z A clinical study on the Korean version of beck anxiety inventory: comparative study of patient and non-patient. Kor J Clin Psychol. (1997) 16:185–97.

[33] Han E Cho Y Park S Kim H Kim S Factor structure of the Korean version of the beck anxiety inventory: an application of confirmatory factor analysis in psychiatric patients. Kor J Clin Psychol. (2003) 22:261–70.

[34] Kwon SM Differential Roles of Dysfunctional Attitudes and Automatic Thoughts in Depression: an Integrated Cognitive Model of Depression. dissertation, Brisbane: University of Queensland (1992).

[35] Faul F Erdfelder E Buchner A Lang AG Statistical power analyses using G^*^ Power 3.1: tests for correlation and regression analyses. Behav Res Methods (2009) 41:1149–60. 10.3758/BRM.41.4.1149 19897823

[36] Faul F Erdfelder E Lang AG Buchner A. G^*^ Power 3: a flexible statistical power analysis program for the social, behavioral, and biomedical sciences. Behav Res Methods (2007) 39:175–91. 10.3758/BF03193146 17695343

[37] Sheehan DV Lecrubier Y Sheehan KH Amorim P Janavs J Weiller E Hergueta T Baker R Dunbar GC. The Mini-International Neuropsychiatric Interview (M.I.N.I.): the development and validation of a structured diagnostic psychiatric interview for DSM-IV and ICD-10. J Clin Psychiatry (1998) 59(Suppl.20):22–33. 9881538

[38] Yoo SW Kim YS Noh JS Oh KS Kim CH Namkoong K Validity of Korean version of the mini-international neuropsychiatric interview. Anxiety Mood (2006) 2:50–5.

[39] Meyer TJ Miller ML Metzger RL Borkovec TD. Development and validation of the penn state worry questionnaire. Behav Res Ther. (1990) 28:487–95. 10.1016/0005-7967(90)90135-6 2076086

[40] Fresco DM Mennin DS Heimberg RG Turk CL. Using the penn state worry questionnaire to identify individuals with generalized anxiety disorder: a receiver operating characteristic analysis. J Behav Ther Exp Psychiatry (2003) 34:283–91. 10.1016/j.jbtep.2003.09.001 14972674

[41] Lim YJ Kim YH Lee EH Kwon SM. The penn state worry questionnaire: psychometric properties of the Korean version. Depress Anxiety (2008) 25:E97–103. 10.1002/da.20356 17823961

[42] Spitzer RL Kroenke K Williams JB Löwe B. A brief measure for assessing generalized anxiety disorder: the GAD-7. Arch Intern Med. (2006) 166:1092–7. 10.1001/archinte.166.10.1092 16717171

[43] Seo JG Park SP. Validation of the generalized anxiety disorder-7 (GAD-7) and GAD-2 in patients with migraine. J Headache Pain (2015) 16:97. 10.1186/s10194-015-0583-8 26596588 PMC4656257

[44] Seo JG Cho YW Lee SJ Lee JJ Kim JE Moon HJ. Validation of the generalized anxiety disorder-7 in people with epilepsy: a MEPSY study. Epilepsy Behav. (2014) 35:59–63. 10.1016/j.yebeh.2014.04.005 24798411

[45] Beck AT Steer RA Brown GK. Beck depression inventory-II. San Antonio (1996) 78:490–8. 12146814

[46] Sung H Kim J Park Y Bai D Lee S Ahn H A study on the reliability and the validity of Korean version of the beck depression inventory-II (BDI-II). J Korean Soc Biol Ther Psychiatry (2008) 14:201–12.

[47] Lim SY Lee EJ Jeong SW Kim HC Jeong CH Jeon TY The validation study of beck depression scale 2 in Korean version. Anxiety Mood (2011) 7:43–53.

[48] Yu B Lee HK Lee K Validation and factor structure of Korean version of the Beck depression inventory second edition (BDI-II): in a university student sample. Korean J Biol Psychiatry (2011) 18:126–33.

[49] Radloff LS. The use of the center for epidemiologic studies depression scale in adolescents and young adults. J Youth Adolesc. (1991) 20:149–66. 10.1007/BF01537606 24265004

[50] Chon KK Choi SC Yang BC Integrated adaptation of CES-D in Korea. Korean Korean J Health Psychol. (2001) 6:59–76.

[51] Cho MJ Kim KH Diagnostic validity of the CES-D (Korean version) in the assessment of DSM-III-R major depression. J Korean Neuropsychiatr Assoc. (1993) 32:381–99.

[52] Kroenke K Spitzer RL Williams JB. The PHQ-9: validity of a brief depression severity measure. J Gen Intern Med. (2001) 16:606–13. 10.1046/j.1525-1497.2001.016009606.x 11556941 PMC1495268

[53] Choi HS Choi JH Park KH Joo KJ Ga H Ko HJ Standardization of the Korean version of patient health questionnaire-9 as a screening instrument for major depressive disorder. J Korean Acad Fam Med. (2007) 28:114–9.

[54] Park SJ Choi HR Choi JH Kim KW Hong JP Reliability and validity of the Korean version of the patient health questionnaire-9 (PHQ-9). Anxiety Mood (2010) 6:119–24.

[55] An J Seo E Lim K Shin J Kim J Standardization of the Korean version of screening tool for depression (Patient Health Questionnaire-9, PHQ-9). J Korean Soc Biol Ther Psychiatry (2013) 19:47–56.

[56] Taylor S Cox BJ. An expanded anxiety sensitivity index: evidence for a hierarchic structure in a clinical sample. J Anxiety Disord. (1998) 12:463–83. 10.1016/S0887-6185(98)00028-0 9801964

[57] Taylor S Zvolensky MJ Cox BJ Deacon B Heimberg RG Ledley DR. Robust dimensions of anxiety sensitivity: development and initial validation of the anxiety sensitivity index-3. Psychol Assess. (2007) 19:176. 10.1037/1040-3590.19.2.176 17563199

[58] Lim YJ Kim JH. Korean anxiety sensitivity index-3: its factor structure, reliability, and validity in non-clinical samples. Psychiatry Investig. (2012) 9:45–53. 10.4306/pi.2012.9.1.45 22396684 PMC3285740

[59] Rapee RM Craske MG Barlow DH. Assessment instrument for panic disorder that includes fear of sensation-producing activities: the albany panic and phobia questionnaire. Anxiety (1994) 1:114–22. 10.1002/anxi.3070010303 9160559

[60] Kim JH Yang JC Kim JB Lim KY Lee SY Yu BH A validation study of Korean albany panic and Phobia Questionnaire (APPQ). J Korean Neuropsychiatr Assoc. (2004) 43:329–36.

[61] Clark LA Watson D. Tripartite model of anxiety and depression: psychometric evidence and taxonomic implications. J Abnorm Psychol. (1991) 100:316. 10.1037/0021-843X.100.3.316 1918611

[62] Park BS Hoe MS Testing factor structure and measurement invariance of the korean version of beck anxiety inventory (BAI) for Korean adolescents. Mental Health Social Work (2016) 44:150–79.

[63] Cox BJ Cohen E Direnfeld DM Swinson RP. Does the beck anxiety inventory measure anything beyond panic attack symptoms? Behav Res Ther. (1996) 34:949–54. 10.1016/S0005-7967(96)00037-X 8990548

[64] Parker G Gladstone G Chee KT. Depression in the planet's largest ethnic group: the Chinese. Am J Psychiatry (2001) 158:857–64. 10.1176/appi.ajp.158.6.857 11384889

[65] Ryder AG Yang J Heine SJ Somatization vs. psychologization of emotional distress: a paradigmatic example for cultural psychopathology. Online Read Psychol Cult. (2002) 10:3 10.9707/2307-0919.1080

[66] Lee K Kim D Cho Y. Exploratory factor analysis of the beck anxiety inventory and the beck depression inventory-ii in a psychiatric outpatient population. J Korean Med Sci. (2018) 33:e128. 10.3346/jkms.2018.33.e128 29651821 PMC5897159

[67] Lai CYY Ho CSH Lim CR Ho RCM Functional near-infrared spectroscopy in psychiatry. BJPsych Adv. (2017) 23:324–30. 10.1192/apt.bp.115.015610

[68] Mathersul D Williams LM Hopkinson PJ Kemp AH. Investigating models of affect: Relationships among EEG alpha asymmetry, depression, and anxiety. Emotion (2008) 8:560–72. 10.1037/a0012811 18729586

[69] Bress JN Meyer A Hajcak G. Differentiating anxiety and depression in children and adolescents: evidence from event-related brain potentials. J Clin Child Adolesc Psychol. (2015) 44:238–49. 10.1080/15374416.2013.814544 23879474

[70] Bruder GE Fong R Tenke CE Leite P Towey JP Stewart JE. Regional brain asymmetries in major depression with or without an anxiety disorder: a quantitative electroencephalographic study. Biol Psychiatry (1997) 41:939–48. 10.1016/S0006-3223(96)00260-0 9110099

[71] Ho CSH Zhang MWB Ho RCM. Optical topography in psychiatry: a chip off the old block or a new look beyond the mind–brain frontiers? Front Psychiatry (2016) 7:74. 10.3389/2Ffpsyt.2016.00074 27199781 PMC4844608

[72] Gibbons RD Weiss DJ Pilkonis PA Frank E Moore T Kim JB. Development of the CAT-ANX: a computerized adaptive test for anxiety. Am J Psychiatry (2014) 171:187–94. 10.1176/appi.ajp.2013.13020178 23929270 PMC4052830

[73] Kessler RC Chiu WT Demler O Walters EE. Prevalence, severity, and comorbidity of 12-month DSM-IV disorders in the national comorbidity survey replication. Arch General Psychiatry (2005) 62:617–27. 10.1001/archpsyc.62.6.617 15939839 PMC2847357

[74] Sartorius N Üstün TB Lecrubier Y Wittchen HU Depression comorbid with anxiety: results from the WHO study on psychological disorders in primary health care. Br J Psychiatry (1996) 168:38–43. 10.1192/S0007125000298395 8864147

[75] Aldao A Nolen-Hoeksema S Schweizer S. Emotion-regulation strategies across psychopathology: a meta-analytic review. Clin Psychol Rev. (2010) 30:217–37. 10.1016/j.cpr.2009.11.004 20015584

[76] Campbell-Sills L Barlow DH Brown TA Hofmann SG. Acceptability and suppression of negative emotion in anxiety and mood disorders. Emotion (2006) 6:587. 10.1037/1528-3542.6.4.587 17144750

[77] Loo CK Mitchell PB. A Review of the Efficacy of Transcranial Magnetic Stimulation (TMS) Treatment for Depression, and Current and Future Strategies to Optimize Efficacy. J Affect Disord. (2005) 88:255–67. 10.1016/j.jad.2005.08.001 16139895

[78] Pagnin D de Queiroz V Pini S Cassano GB. Efficacy of ECT in depression: a meta-analytic review. J ECT (2004) 20:13–20. 10.1097/00124509-200403000-00004 15087991

[79] Barlow DH Anxiety and its Disorders: The Nature and Treatment of Anxiety and Panic. Guilford Publications New York, NY (2013).

[80] Stahl SM Stahl SM Stahl's Essential Psychopharmacology: Neuroscientific Basis and Practical Applications. Cambridge: Cambridge University Press (2013).

[81] Whiting PF Rutjes AW Westwood ME Mallett S Deeks JJ Reitsma JB. QUADAS-2: a revised tool for the quality assessment of diagnostic accuracy studies. Ann Intern Med. (2011) 155:529–36. 10.7326/0003-4819-155-8-201110180-00009 22007046

